# A Test for Tea

**Published:** 1889-08

**Authors:** 


### A TEST FOR TEA.

A Russian analyst gives the following as a test by which tea can be proved to be genuine or not : Take a pinch of tea in a glass, pour upon it a little cold water and shake it up well. Pure tea will only slightly color the water, while a strong infusion is quickly got from the adulterated or painted leaf. Now boil both sorts separately, and let them stand till oool, and the difference betheen them will be most marked. The false tea will become still stronger after a long standing, but will remain transparent. Whereas pure tea will become muddy or milky. This last apperrance arises from the tannic acid, which is a natural property in pure tea, but which in artificial tea is entirely absent.

